# Preparation and Characterization of Novel Gellan Gum Hydrogels Suitable for Modified Drug Release

**DOI:** 10.3390/molecules14093376

**Published:** 2009-09-03

**Authors:** Pietro Matricardi, Claudia Cencetti, Roberto Ria, Franco Alhaique, Tommasina Coviello

**Affiliations:** Department of Drug Chemistry and Technologies, Faculty of Pharmacy, University of Rome “La Sapienza”, Piazzale Aldo Moro 5, 00185, Rome, Italy; E-mails: pietro.matricardi@uniroma1.it (P.M.); claudia.cencetti@fastwebnet.it (C.C.); roberto.ria@hotmail.it (R.R.); franco.alhaique@uniroma1.it (F.A.)

**Keywords:** Gellan, hydrogels, modified drug release, rheology, texture analysis

## Abstract

Innovative hydrogels obtained by physical and chemical crosslinking of deacylated Gellan gum have been characterized in terms of water uptake, rheological properties and compressibility, and the behaviour of the tested materials, according to the type of the obtained network, is thoroughly discussed. The release from the various gels of loaded model molecules of different steric hindrance was also investigated and the trend of the release profiles has been related to the structures proposed for the physical and the chemical hydrogel.

## 1. Introduction

Over the past decades, great attention was been focused on biopolymer-based hydrogels for use as potential carriers in controlled drug delivery [1,2]. Hydrogels are three-dimensional, hydrophilic, polymeric networks capable of imbibing large amount of water or biological fluids [3], resembling biological tissues. Because of this property, great interest was devoted to these systems for biomedical applications. Indeed, the physicochemical properties of the hydrogels can be tuned by varying the crosslinking degree (physical and/or chemical), thus making these networks suitable devices for a modulated drug delivery.

Deacylated Gellan gum (Gellan) is an anionic microbial polysaccharide, secreted from *Sphingomonas elodea*, consisting of repeating tetrasaccharide units of glucose, glucuronic acid and rhamnose residues in a 2:1:1 ratio: [→3)–β–D–glucose–(1→4)–β–D–glucuronic acid–(1→4)–β–D–glucose–(1→4)–α–L–rhamnose–(1→]. In the native polymer two acyl substituents, L-glyceryl at O(2) and acetyl at O(6), are present on the 3-linked glucose. On average, there is one glyceryl per repeating unit and one acetyl for every two repeating units. Deacylated Gellan gum is obtained by alkali treatment of the native polysaccharide.

Both native and deacylated Gellan gum are capable of physical gelation [4]. To induce Gellan gelation it is necessary to warm up preliminarily a concentrated water solution of the polysaccharide: when the temperature is decreased, the chains undergo a conformational transition from random coils to double helices (Coil-Helix Transition). Then a rearrangement of the double helices occurs leading to the formation of ordered junction zones (Sol-Gel Transition) [5] thus giving a thermo-reversible hydrogel [6]. Much stronger physical thermo-reversible hydrogels are also obtained by addition of mono and divalent ions to Gellan solutions [7], or in acidic conditions [8].

The physical gelation properties make this polysaccharide suitable as a structuring and gelling agent in food industries. Gellan is also exploited in the field of modified release of bioactive molecules. Aqueous solutions of Gellan are used as *in situ* gelling systems, mainly for ophthalmic preparation [8] and for oral drug delivery [9]. Physical Gellan hydrogels, prepared with mono or divalent cations, are used also for the preparation of tablets, beads [10] or microspheres. Interpenetrating polymer networks [11] or co-crosslinked polymer networks [12] based on Gellan and other polysaccharide systems have also been developed as drug delivery matrices.

Chemical hydrogels of Gellan are usually prepared via chemical crosslinking of preformed physical networks, in order to enhance their mechanical properties, and to obtain slower drug release profiles [13]. The aim of the present work was the development of a novel Gellan chemical hydrogel, with tunable physicochemical properties, obtained by crosslinking the polymer chains with L-lysine ethyl ester moieties. As a first step, amidation of Gellan carboxyl groups in the presence of 1-ethyl-3-(3-dimethylaminopropyl)carbodiimide (EDC) and *N*-hydroxysuccinimide (NHS) was carried out [14,15]: it is well known that EDC and NHS have the ability to mediate the amide bond formation between amino and carboxyl groups. Ethyl ester of L-lysine (Lys) was used in order to protect the carboxyl group, thus avoiding the intermolecular reaction among the L-lysine molecules. Chemical hydrogels with different crosslinking degrees were prepared, and their physico-chemical and rheological properties were studied and compared with the corresponding physical gels. The new networks, that were also investigated as matrices for modified oral release, using model molecules with different steric hindrance, can be proposed as carriers for the delivery of high molecular weight drugs such as proteins. Furthermore, the observed peculiar mechanical and rheological properties can be properly modulated in order to give matrices suitable for depot systems.

## 2. Results and Discussion

Experiments were carried out on different types of hydrogels:

- physical gel of Gellan (cooling of hot solutions)
- physical gel of Gellan in the presence of different amounts of Lys (cooling of hot solutions)
- chemical gel of Gellan in the presence of different amounts of Lys and EDC/NHS

### 2.1. Structural differences between physical and chemical hydrogels

The swelling properties, the rheological and dynamomechanical characterization and the release profiles of various model molecules evidenced the great difference between physical and chemical hydrogels due to the structural differences of the tested materials.

**Figure 1:**
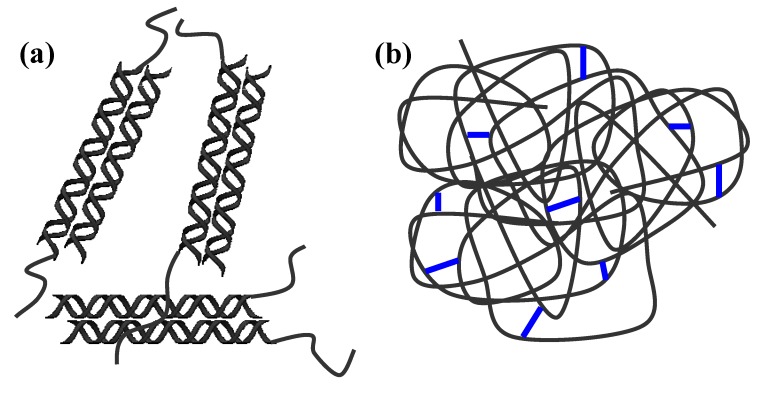
Schematic representation of: (a) physical gel of Gellan; (b) chemical gels of Gellan.

When Gellan is solubilized at high temperatures (T ≥ 80 °C) the polymer chains are in a random coil conformation. The physical gels, in the presence or without Lys, are obtained, as reported above, by cooling the hot solution: the random chains rearrange in a “double helix” conformation (coil-helix transition), and the double helixes assemble leading to physical junction zones (sol-gel transition) (Figure 1-a), whose stability depends on the solution composition (presence of mono or bivalent cations, pH value, etc.).

For the preparation of the chemical gels EDC and NHS were added to the hot solution of Gellan (T ≈ 50 °C). The crosslinking occurs between the carboxyl groups of Gellan polymer chains, in random coil conformation, and the amine groups of Lys. Thus, the disordered structure of the polymeric chains is maintained by the chemical linkages also when the temperature is lowered to room temperature (Figure 1-b).

According to the proposed structure, the physical hydrogels should have a quite low water uptake (because the carboxylic groups are involved in the formation of the double helices) and, at the same time, a relevant elastic behaviour, which increases with the amount of the junction zones.

On the other hand, chemical hydrogels, due to the presence of disordered chains, should have a high water uptake (inversely proportional to the crosslinking degree) and, correspondingly, a small elastic behaviour.

### 2.2. Water uptake

**Figure 2:**
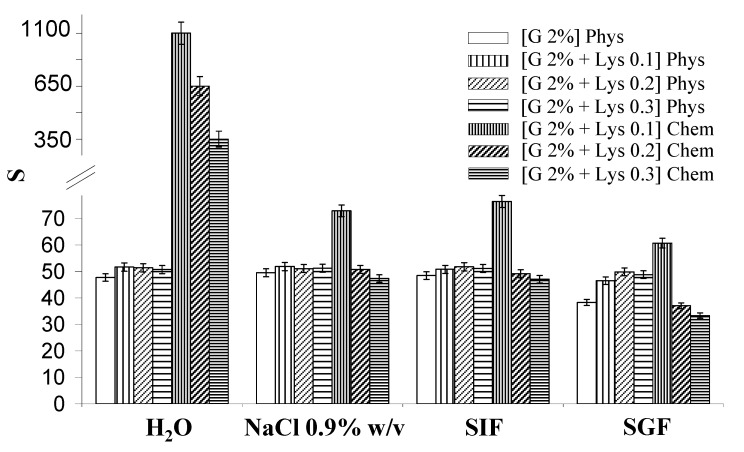
Values of water uptake parameter (S) for the various hydrogels swollen in different media at 37 °C. The data are presented as arithmetic mean of three different measurements, ± SD. (in the legend G = Gellan).

The water uptake capability is an important parameter for materials to be used in biomedical applications. Water uptake was evaluated, in different media, for the Gellan physical and chemical hydrogels. Results are reported in Figure 2 (S values).

The physical hydrogels show a similar behaviour in the different media, meaning that their different composition does not affect their water uptake: only the physical hydrogel [Gellan 2%] show slightly lower values of the parameters S and w_R_, especially in SGF (Simulated Gastric Fluid, HCl 0.1 M, pH = 1) solution. This behaviour is probably due to the low pH environmental conditions, which induce the formation of more stable junction zones. In fact, at pH = 1, a suppression of the polyelectrolytic behaviour of Gellan chains occurs, because the carboxylate groups are in their acidic form. In these conditions the polymer chains can be closer one another leading to the macroscopic shrinking of the gel.

For the chemical crosslinked samples the water uptake is strongly dependent both on hydrogel and solution compositions. As expected, water uptake is inversely proportional to the crosslinking degree of the sample. When the hydrogels are swollen in water, their behaviour is quite similar to that of the super-absorbent materials [16], showing very high values of the swelling parameter (e.g., S = 350 for the [Gellan 2% + Lys 0.3]_Chem_, and S = 1,070 for the [Gellan 2% + Lys 0.1]_Chem_).

In physiological solution (NaCl 0.9% w/v) and in SIF (Simulated Intestinal Fluid, Phosphate Buffer, pH = 7.4) the crosslinked and physical hydrogels show low S values, similar to those of the Gellan gum hydrogels, due to the presence of a large amount of Na^+^ counterions that reduce the electrostatic repulsions among the carboxylic groups of the polymeric chains, leading to a more compact overall structure. The sample [Gellan 2% + Lys 0.1]_Chem_ represents an exception to this general behaviour: in this case the water uptake is significantly higher, because the polymer chains are in a random coil conformation (as depicted in the model) and the network, with a quite low crosslinking degree, is able to absorb high amounts of water. Again, in SGF, a reduced water uptake is observed. Similar comments can be made about the w_R_ parameter (see Table 1).

**Table 1:** w_R_ ratio (w_eq_/w_0_) for the different hydrogels, swollen in different media at 37 °C. The data are presented as arithmetic mean of three different measurements ± SD.

	H_2_O	NaCl 0.9% w/v	SIF	SGF
**[Gellan 2%]_Phys_**	0.96 ± 0.03	0.99 ± 0.04	0.97 ± 0.02	0.77 ± 0.02
**[Gellan 2% + Lys0.1]_Phys_**	1.03 ± 0.04	1.04 ± 0.03	1.02 ± 0.04	0.93 ± 0.02
**[Gellan 2% + Lys 0.2]_Phys_**	1.02 ± 0.02	1.02 ± 0.04	1.03 ± 0.03	1.00 ± 0.04
**[Gellan 2% + Lys 0.3]_Phys_**	0.99 ± 0.02	1.02 ± 0.02	1.02 ± 0.04	0.98 ± 0.02
**[Gellan 2% + Lys 0.1]_Chem_**	21.95 ± 0.20	1.48 ± 0.03	1.54 ± 0.03	1.34 ± 0.04
**[Gellan 2% + Lys 0.2]_Chem_**	12.98 ± 0.15	1.04 ± 0.02	1.02 ± 0.03	0.75 ± 0.03
**[Gellan 2% + Lys 0.3]_Chem_**	7.06 ± 0.15	0.99 ± 0.04	0.98 ± 0.02	0.70 ± 0.02

### 2.3. Rheological measurements

**Figure 3:**
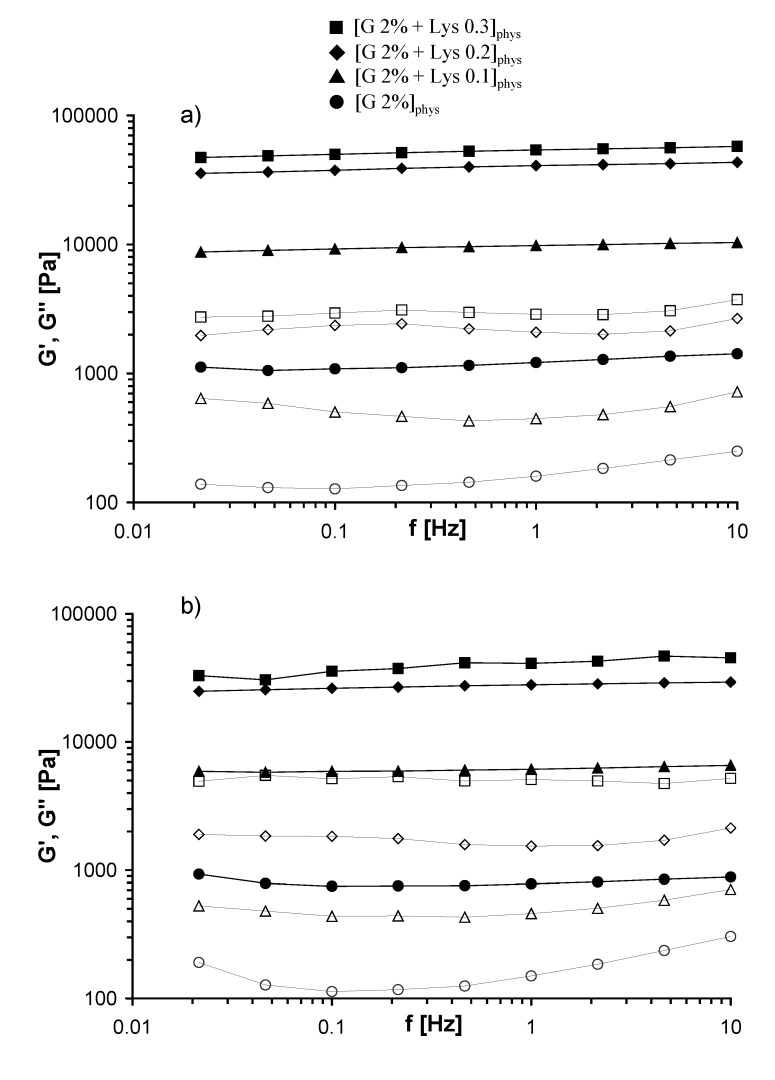
Frequency Sweep of Gellan physical gels, performed at 25 °C (a) and 37 °C (b). G’ (filled symbols), G’’ (empty symbols) (in the legend G = Gellan).

Mechanical spectra of the different Gellan physical hydrogels, recorded at 25 and 37 °C, are reported in Figure 3: a typical gel behaviour is clearly evidenced, with the storage modulus, G’, and the loss modulus, G’’, almost independent on the frequency and with G’ higher of at least of a factor ten with respect to G”.

G’ values obtained at 37 °C are smaller (about 25-30 %) than those obtained at 25 °C, and G’’ values remain almost constant: this behaviour is due to the thermoreversible nature of the physical hydrogels. As a matter of fact, at room temperature the junction zones between adjacent helices are more stable and stronger; when the temperature increases the number of junction zones should decrease leading to a weaker gel (the elastic moduli decrease).

**Figure 4:**
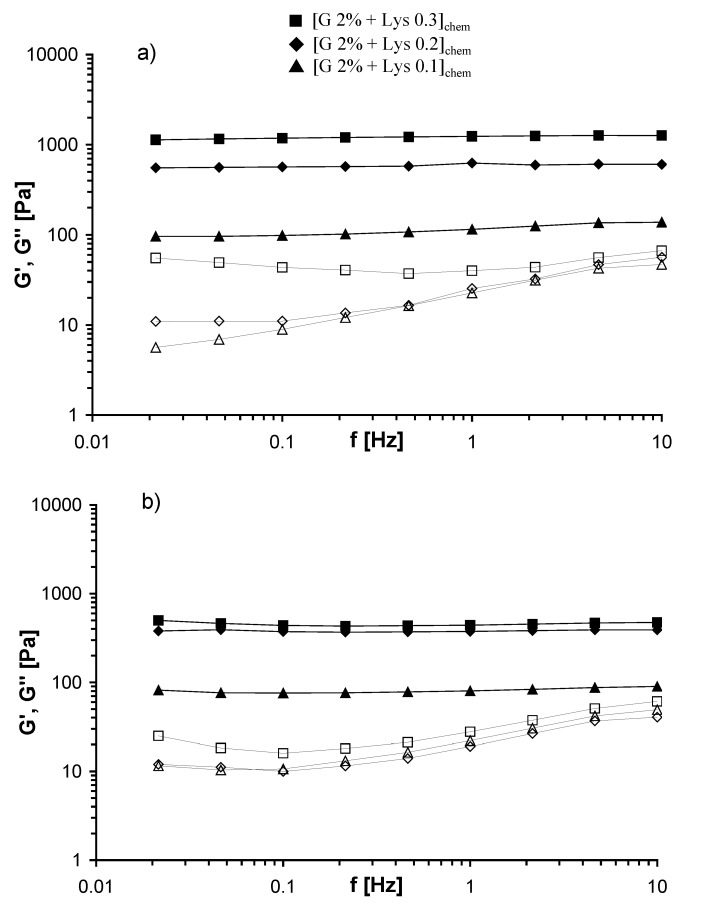
Frequency Sweep of Gellan chemical gels, performed at 25 °C (a) and 37 °C (b). G’ (filled symbols), G’’ (empty symbols) (in the legend G = Gellan).

**Figure 5:**
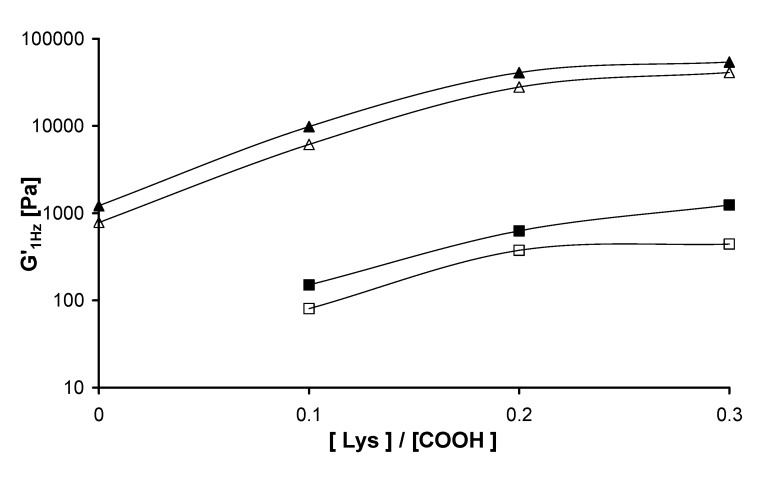
G’(1 Hz) at 25 °C (full symbols) and 37 °C (empty symbols) for the chemical Gellan hydrogel (squares) and physical Gellan hydrogels (triangles).

Mechanical spectra of the Gellan chemical hydrogels, measured at 25 and 37 °C, are reported in Figure 4: also in this case typical gel behaviour is evidenced. Furthermore, also in this case the increase of the temperature leads to weaker hydrogels, with G’ values at 37 °C smaller in comparison with those obtained at 25 °C. G’ values of the chemical gels are much smaller (more than two orders of magnitude) than those of the corresponding physical gels for both temperatures (see Figure 5).

This behaviour can be related to the organization of different Gellan chains in the physical and in the chemical hydrogels: in the chemical gel the presence of chains still in the disordered conformation, and therefore quite “free to flow”, gives a significant contribution to the dissipative modulus (G”) (see Figure 1b). As evidenced in Figure 5, as Lys content increases G’ values (1 Hz) increase for both the chemical and the physical gel. In the former case, because more chemical crosslinkings are formed; in the second case, because the addition of Lys reduces the electrostatic repulsions among the polysaccharidic chains, thus favoring the formation of a higher number of junction zones [8]. The trend of G’ is almost the same when the experiments were carried out at 25 and 37 °C; thus indicating that the network structure is not significantly affected by this temperature variation.

The frequency sweep measurements were carried out in the linear viscoelastic regime. Furthermore, measurements in large shear deformation were also performed in order to study the behaviour of the prepared physical and chemical hydrogels in such stressed conditions. In particular, yield stress (σ_Yield_) and yield deformation (γ_Yield_) data were collected at 25 and 37 °C (see Table 2).

**Table 2:** Values of yield stress (σ_Yield_) and yield deformation (γ_Yield_) for the different hydrogels, obtained at 25 °C and 37 °C.

	T = 25 °C		T = 37 °C	
	σ_Yield_ (Pa)	γ_Yield_	σ_Yield_ (Pa)	γ_Yield_
**[Gellan 2% + Lys 0.1]_Phys_**	2226 ± 509	0.335 ± 0.218	1646 ± 110	0.324 ± 0.003
**[Gellan 2% + Lys 0.2]_Phys_**	2457 ± 1034	0.094 ± 0.040	2587 ± 698	0.203 ± 0.061
**[Gellan 2% + Lys 0.3]_Phys_**	3296 ± 146	0.095 ± 0.003	3235 ± 76	0.145 ± 0.011
**[Gellan 2% + Lys 0.1]_Chem_**	1130 ± 34	11.585 ± 0.417	701 ± 88	2.705 ± 0.358
**[Gellan 2% + Lys 0.2]_Chem_**	1210 ± 314	2.160 ± 0.452	675 ± 50	1.782 ± 0.075
**[Gellan 2% + Lys 0.3]_Chem_**	1321 ± 160	1.425 ± 0.219	770 ± 290	0.729 ± 0.080

For the physical hydrogels σ_Yield_ values increase with Lys content, while γ_Yield_ values decrease: such behaviour is in agreement with the information acquired by the mechanical spectra. When the temperature increases the values of σ_Yield_ and γ_Yield_ are of the same order of magnitude of those obtained at 25 °C and the same trend is confirmed.

Also for the chemical hydrogels, at both temperatures, the same trend is observed: with the increasing of crosslinking degree, σ_Yield_ increase and γ_Yield_ decrease. It must be pointed out that the chemically crosslinked samples show smaller σ_Yield_ values and significantly higher γ_Yield_ values, in comparison to the corresponding physical hydrogels.

### 2.4. Compressibility tests

The results obtained at 25 °C by means of dynamomechanical tests are reported in Table 3.

**Table 3:** Values of the area under the σ_N_ = f(γ) curve, A1, compressibility, A3/A1, and Young Modulus, E for the tested hydrogels (25 °C).

	A1 (N/m^2^)	A3/A1 (%)	E (N/m^2^)
**[Gellan 2% + Lys 0.1]_Phys_**	38950 ± 1530	65.6 ± 0.5	73830 ± 3740
**[Gellan 2% + Lys 0.2]_Phys_**	72890 ± 17800	72.8 ± 3.7	154690 ± 38000
**[Gellan 2% + Lys 0.3]_Phys_**	74100 ± 19400	87.4 ± 0.5	168090 ± 38290
**[Gellan 2% + Lys 0.1]_Chem_**	10990 ± 1660	99.1 ± 0.6	13440 ± 1470
**[Gellan 2% + Lys 0.2]_Chem_**	14100 ± 2130	94.2 ± 0.8	18470 ± 4750
**[Gellan 2% + Lys 0.3]_Chem_**	17920 ± 3140	88.1 ± 1.2	24990 ± 5700

The values of Young Modulus (E) confirm the results of rheological measurements: for the physical hydrogels, E increases with Lys content; for the chemical hydrogels, E values increase with the crosslinking degree, even if the absolute values are much smaller than those calculated for the corresponding physical samples.The compressibility tests show, for the physical hydrogels, an increase of the A3/A1 value with the amount of Lys. For the chemical hydrogel, the values of A3/A1 are greater than those observed with physical sample, but these values decrease with the crosslinking degree. Again, this behaviour can be related to the structural differences between the networks: in the physical hydrogels, the increase of junction zones (due to the increasing Lys content) leads to a more elastic and compressible network. On the other side, chemical networks show higher compressibility values, because during the compression those chains that are still in random coil conformation (unlike the double helices) can more efficiently withstand the compression. This capability “to flow” decreases with the increase of the crosslinking degree, due to the fact that less disordered chains are present in the chemical matrix.

### 2.5. In vitro release studies

#### 2.5.1. Release of vitamin B12

**Figure 6:**
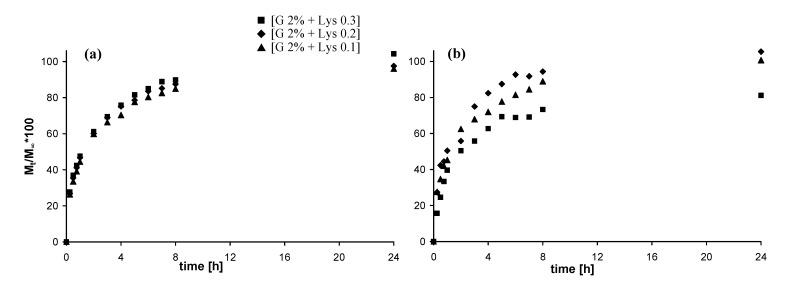
Release profiles (37 °C, SIF) of Vitamin B12: (a) from physical hydrogels; (b) from chemical hydrogels (in the legend G = Gellan).

In Figure 6 the release of Vitamin B12 from the different hydrogels is reported. The release from physical hydrogels is quite fast and more than 80% of the loaded model drug is released after 8 hours. The release profiles of the physical hydrogels (Figure 6-a) are not appreciably affected by the different Lys content. On the other side, the three chemical hydrogels (Figure 6-b) show slightly different profile of release according to the crosslinking degree, i.e. delivery rate decreases with Lys content increases.

#### 2.5.2. Release of fluorescein isothiocyanate-dextran

**Figure 7:**
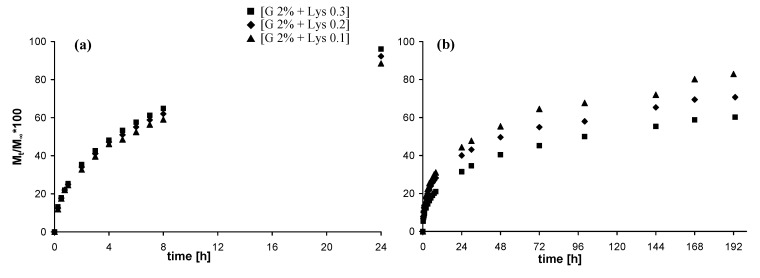
Release profiles (37 °C, SIF) of DexFluo_70_: **(a)** from physical hydrogels; **(b)** from chemical hydrogels (in the legend G = Gellan).

In Figure 7 the release of DexFluo_70_ from the different hydrogels is reported. Due to its high molecular weight, and consequently to its high steric hindrance, release rates are quite slower than those of vitamin B12 [17,18].

The release from the physical hydrogels is much faster then that from the chemical hydrogels: more than 60% of the loaded model molecule is released after 8 hours and almost all the DexFluo_70_ is delivered after 24 hours. Again, the Lys content does not affect the release profiles. The chemical hydrogels show a slower and quite different delivery rate, and even after 8 days, the release of DexFluo70 is not complete. As in the case of Vitamin B12 the crosslinking degree affect the diffusion out of the matrix of the guest molecule 

## 3. Experimental Section

### 3.1. Materials

Deacylated Gellan gum Kelcogel CG-LA (Gellan) was purchased from Kelko: according to the manufacturer, more than 90% of the repeating units are in the sodium salt form. *N*-(3-Dimethylaminopropyl)-*N′*-ethylcarbodiimide hydrochloride (EDC), 2-(*N*-morpholino)ethanesulfonic acid monohydrate (MES), L-lysine ethyl ester dihydrochloride (Lys) and vitamin B12 were purchased from Fluka. *N*-Hydroxysuccinimide (NHS) and fluorescein isothiocyanate-dextran, M_w_ = 59,000-77,000 (FluDex_70_), were purchased from Sigma-Aldrich. All other chemicals were analytical grade, obtained from Sigma and Fluka and used without further purification.

### 3.2. Physical hydrogel preparation

For the preparation of the Gellan physical hydrogels 60 mg of Gellan (89.1 μmol COOH) were dissolved in 2.9 ml of MES buffer (50 mM, pH = 4.0). The temperature of the suspension was first raised up to 80 °C until complete dissolution of the polymer and then cooled to about 50 °C. Then 2.2, 4.4 and 6.6 mg of Lys (8.91, 17.82, 26.73 μmol), dissolved in 0.1 mL MES buffer, were added, and the polymer solution was left to room temperature overnight, until complete formation of the crosslinked hydrogels.

The obtained hydrogels were named [Gellan 2% + Lys 0.1]_Phys_, [Gellan 2% + Lys 0.2]_Phys_ and [Gellan 2% + Lys 0.3]_Phys_ respectively. By a similar procedure, a physical hydrogel of Gellan alone was also prepared, dissolving 60 mg of Gellan in 3.0 mL of MES buffer: the obtained hydrogel was named [Gellan 2%]_Phys_. The prepared hydrogels were used without further purification.

### 3.3. Chemical hydrogel preparation

**Scheme 1:**
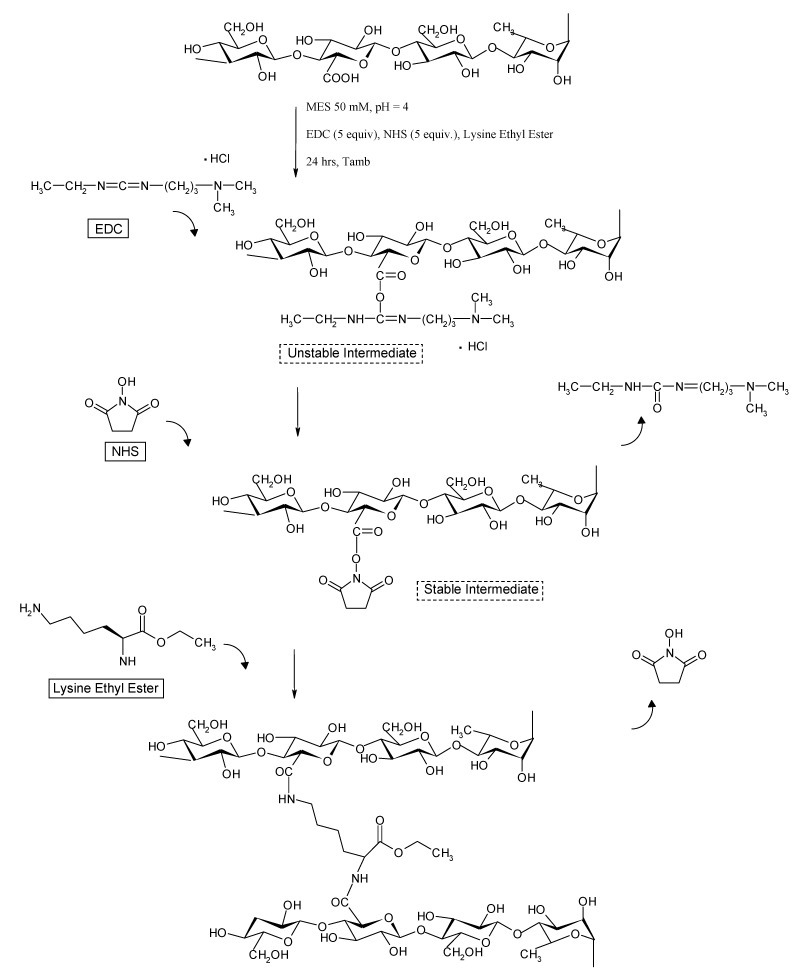
Scheme of the chemical EDC/NHS crosslinking between Gellan and lysine ethyl ester.

The crosslinked hydrogels were prepared using the well known EDC/NHS reaction [14,15], shown in Scheme 1, using the following concentrations and molar ratios:

c_P_ = 20 mg/mL
EDC] / [COOH] = 5
EDC] / [NHS] = 1
Lys] / [COOH] = 0.1, 0.2, 0.3

Gellan (89.1 μmol COOH, 60 mg) was dispersed in MES buffer (2.7 mL, 50 mM, pH = 4.0). The temperature of the suspension was first raised up to 80 °C until complete dissolution of the polymer and then cooled to about 50 °C. EDC (85.4 mg, 445.5 μmol) and NHS (51.3 mg, 445.5 μmol), both dissolved in MES buffer (0.1 mL) were added to the polymer solution. After stirring for a few seconds, 2.2, 4.4 and 6.6 mg of Lys (8.91, 17.82, 26.73 μmol), dissolved in MES buffer (0.1 mL), were added, and the polymer solution was left to room temperature overnight, until complete formation of the crosslinked hydrogels. The obtained hydrogels were named [Gellan 2% + Lys 0.1]_Chem_, [Gellan 2% + Lys 0.2]_Chem_ and [Gellan 2% + Lys 0.3]_Chem_ respectively; the prepared hydrogels were used with no further purification.

### 3.4. Water uptake

The ability of a hydrogel to absorb aqueous solutions was evaluated according to the following equation:

S = (w_eq_ - w_dry_) / w_dry_ (1)

where w_eq_ represents the weight of the hydrogel detected after 24 hours of swelling in different media (*i.e.,* when no further swelling was detected) and w_dry_ represents the weight of the same sample after freeze-drying.

The relative weight of the hydrogel, w_R_, was evaluated according to the following equation:

w_R_ = w_24h_ / w_0_ (2)

where w_0_ represents the weight of the hydrogel as soon as it was prepared.

The hydrogels were swelled in different media, respectively distilled water, physiological solution (NaCl 0.9 % w/v), Simulated Intestinal Fluid (SIF, phosphate buffer, pH = 7.4) and Simulated Gastric Fluid (SGF, HCl 0.1 M, pH = 1). All experiments were carried out in triplicate and the results are reported as arithmetic mean ± SD.

### 3.5. Rheological measurements

Rheological experiments were carried out by means of a controlled stress Haake RheoStress 300 Rotational Rheometer, equipped with a Haake DC10 thermostat. The geometry used for the tests was a cross-hatch plate device (Haake PP35 TI: diameter = 35 mm), in order to reduce the extent of the wall slippage phenomena [19].

The hydrogels, with a thickness of about 1.0–2.0 mm, were removed with the aid of a small spatula from the petri dish where they were prepared and were laid with care on the lower plate of the rheometer. The upper plate was then lowered until it reached the hydrogel surface.

Frequency sweep experiments were performed on the hydrogels at 25 and 37 °C in the range 0.01–10 Hz, in the linear viscoelastic region, preliminary assessed by stress sweep experiments. The mechanical spectra were then recorded applying a constant deformation (γ = 0.01) in the linear regime.

Stress sweep experiments were performed (25 °C and 0.1 Hz) in the range 0.01–30 Pa for the chemical hydrogels, and in the range 0.01–500 Pa for the physical hydrogels.

**Figure 8:**
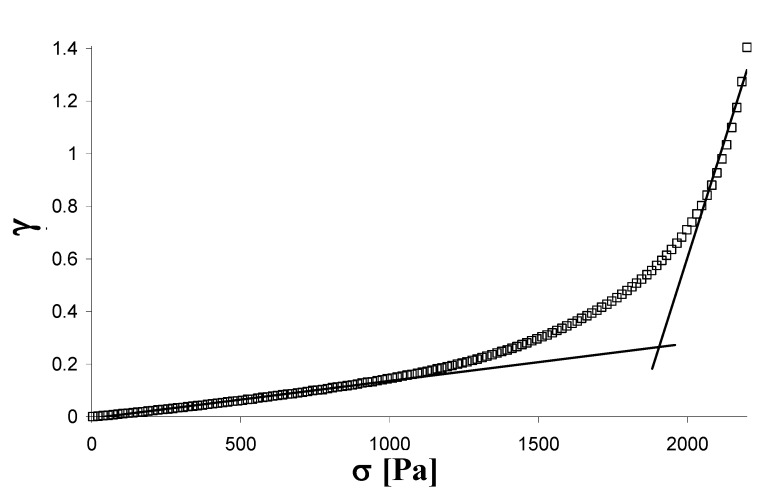
Example of a Yield Stress Test, performed on the physical hydrogel [Gellan 2% + Lys 0.1]_Phys_.

The measurements of yield stress were carried out (25 and 37 °C) applying a linearly increasing stress (0.1–4,000 Pa in 4 minutes) to the hydrogels and recording the resulting deformation γ = f (σ). The yield stress was calculated as the intersection of the lines extrapolated from the linear parts of the experimental curve (Figure 8). All experiments were carried out in triplicate and the calculated values are reported as arithmetic mean ± SD.

### 3.6. Compressibility measurements

A software-controlled dynamometer, TA-XT*2i* Texture Analyzer (Stable Micro Systems, UK), equipped with a 5 kg load cell, was used. The measurements were carried out in three steps: a first compression of the hydrogels (10% of deformation) followed by a second one (25% of deformation) and then by a third compression (10% of deformation). The samples were tested at room temperature, using a steel cylinder probe (P/35, diameter of 35 mm). The pre-test speed, the test speed and the post-test speed were set up at 2, 1 and 2 mm/s, respectively; the trigger force was set up at 0.098 N. The hydrogel samples height was always in the range 2.0–2.5 cm.

Stress-Deformation curves, σ_N_ = f (γ), were obtained, where:

σ_N_ = F/A (N/m^2^) (3)

γ = [(h_0_ -h)/h_0_] × 100 (4)

h_0_ = initial height of the sample, h = final sample height, F = compression force (N) and A = original cross-section area of the hydrogel (m^2^)

The Young’s modulus (E) was determined as the ratio:

E = σ_Max_/γ_10%_ (5)

where σ_Max_ is the value of σ_N_ for γ = 10%, obtained in the first cycle of compression.

**Figure 9:**
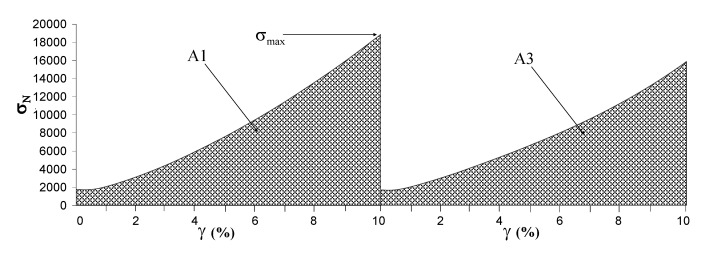
Stress-Deformation curves for the 1^st^ and 3^rd^ compression. σ_max_ = σ_N_ (γ = 10%), A1, A3 = areas under the stress-deformation curves (γ = 0–10%).

The compressibility of the hydrogels was calculated as the ratio of the areas A3/A1, where A3 is the area under the stress-deformation curve for the third compression (10% of deformation) and A1 is the corresponding area for the first compression (10% of deformation) (see Figure 9).

All experiments were repeated at least six times and the results are reported as arithmetic mean ± SD.

### 3.7. In vitro release

For the in vitro release, vitamin B12 and fluorescein isothiocyanate-dextran (FluDex_70_) were used as model molecules. These substances were added during the hydrogel preparation to a final concentration of 1-2 mg/mL. The loaded hydrogels (3 mL, c_P_ = 2% w/v) were kept at a certain height from the bottom of a container by a thin web, while the dissolution medium (200 mL of SIF) was gently magnetically stirred at 37 °C. The solutions were kept under constant magnetic stirring. Aliquots of the SIF solution (5 mL) were taken at regular time intervals and replaced by an equal volume of fresh buffer; the reported release data take into account the dilution effect. The concentration of vitamin B12 was determined with a Perkin-Elmer UV-Vis spectrophotometer LAMBDA 25 using a quartz cell (pathlength 1.0 cm) at λ = 361 nm. The concentration of FluDex_70_ was determined with a Perkin-Elmer Fluorescence Spectrometer LS 50B equipped with a quartz cell (pathlength 1.0 cm) (λ_Exc_ = 495 nm, Slit 2.5 mm; λ_Em_ = 520 nm, Slit 2.5 mm; integration time = 5 sec). All the measurements were performed in triplicate and the obtained values always lay within 10% of the mean.

## 4. Conclusions

Gellan polysaccharide has been used to prepare novel physical and chemical hydrogels and the differences between the two types of gels were evaluated. The physical networks were much stronger than the chemical ones due to the formation of very tight junction zones whose strength depends on the Lys content. On the other side, the chemical gels were weaker because of the applied crosslinking procedure: in fact the crosslinker was added when the polymeric chains were in a disordered conformation and therefore junction zones formation was forbidden. As a final effect the elastic modulus was lower of two orders of magnitude in comparison to those recorded for the physical networks. Of course, within the same kind of gel, physical or chemical ones, the storage modulus increased with the increase of the Lys content. In the physical gels the role of Lys was to decrease the electrostatic repulsions among the chains and therefore the number of the junction zones was a function of the Lys content. In the chemical gel, obviously, the degree of crosslinking increases with the increase of Lys content. According to this model, also the yield stress and the compressibility parameters were affected by the kind and the degree of linkage among the polysaccharidic chains.

The structural differences also influenced the delivery profiles, in particular when high molecular weight model molecules were used. Lys content does not affect significantly the delivery rate from the physical hydrogels, regardless of the steric hindrance of the guest molecules, due to the temporary nature of the junction zones. On the other side, for the chemical gels, quite different profiles were recorded that were deeply influenced by the steric hindrance of the model molecule as well as by the Lys content and a significant amount of the model molecule with the highest molecular weight (DexFluo_70_) still remains entrapped within the network even after 8 days.
